# Abnormal spectral and scale-free properties of resting-state EEG in girls with Rett syndrome

**DOI:** 10.1038/s41598-023-39398-7

**Published:** 2023-08-09

**Authors:** Olga Sysoeva, Vladimir Maximenko, Alexander Kuc, Victoria Voinova, Olga Martynova, Alexander Hramov

**Affiliations:** 1 Center for Cognitive Sciences, Sirius University of Science and Technology, Sochi, Russia 354340; 2 Institute of Higher Nervous Activity and Neurophysiology, Russian Academy of Sciences, Butlerova St. 5a, Moscow, Russia 117485; 3 Artificial Intelligence and Neurotechnology Lab, Privolzhsky Research Medical University, Nizhny Novgorod, Russia 603950; 4 Baltic Center for Artificial Intelligence and Neurotechnology, Immanuel Kant Baltic Federal University, A. Nevskogo Str., Kaliningrad, Russia 236016; 5 Veltischev Research and Clinical Institute for Pediatrics of the Pirogov, Russian National Research Medical University, Ministry of Health of Russian Federation, Moscow, Russia 125412; 6 Mental Health Research Center, Moscow, Russia 117152

**Keywords:** Autism spectrum disorders, Biomarkers, Neural circuits

## Abstract

Spontaneous EEG contains important information about neuronal network properties that is valuable for understanding different neurological and psychiatric conditions. Rett syndrome (RTT) is a rare neurodevelopmental disorder, caused by mutation in the *MECP2* gene. RTT is characterized by severe motor impairments that prevent adequate assessment of cognitive functions. Here we probe EEG parameters obtained in no visual input condition from a 28-channels system in 23 patients with Rett Syndrome and 38 their typically developing peers aged 3–17 years old. Confirming previous results, RTT showed a fronto-central theta power (4–6.25 Hz) increase that correlates with a progression of the disease. Alpha power (6.75–11.75 Hz) across multiple regions was, on the contrary, decreased in RTT, also corresponding to general background slowing reported previously. Among novel results we found an increase in gamma power (31–39.5 Hz) across frontal, central and temporal electrodes, suggesting elevated excitation/inhibition ratio. Long-range temporal correlation measured by detrended fluctuation analysis within 6–13 Hz was also increased, pointing to a more predictable oscillation pattern in RTT. Overall measured EEG parameters allow to differentiate groups with high accuracy, ROC AUC value of 0.92 ± 0.08, indicating clinical relevance.

## Introduction

Spontaneous electrical activity of the brain (EEG, electroencephalogram) contains information about human state and individuality. EEG as a direct measure of postsynaptic currents can be used to non-invasively dig into the neurophysiological processes’ dynamics with milliseconds resolution. Traditionally, EEG spectra are divided into subbands with different functional properties (delta: < 4 Hz, theta: 4–7 Hz, alpha: 8–13 Hz, beta: 14–30 Hz and gamma: > 30 Hz), however these boundaries are rather artificial. In general, the slower the oscillation the larger the population of neurons it can engage. Different oscillations from different regions overlap and interact, reflecting neurophysiological and psychological processes^1,2^. Recently, new properties of spontaneous EEG were described and became increasingly popular. For example, it was shown that EEG exhibits scale-free temporal patterns, including slowly decaying autocorrelation, called long-range temporal correlation (LRTC^3^). LRTC is suggested to reflect the ability of neural networks to integrate information over relatively long time intervals.

Spontaneous EEG is often used in clinics as an additional tool for diagnostic purposes as well as to define optimal treatment plans. For example, many neurodevelopmental disorders are characterized by increased low-frequency power^4,5^. At the same time, particular combinations of different properties of resting EEG can point to specific neurophysiological abnormalities and allow differential diagnosis^6^.

Rett Syndrome (RTT) is the rare neurodevelopmental disorder characterized by poor motor and cognitive skills that is also hard to assess due to lack of speech and problems with voluntary movements^7^. RTT is characterized by initially normal development followed by regression usually occurring within the first three years of life. After this period the condition can be relatively stable. Most cases of this disorder are attributed to *MECP2* gene dysfunction and is found in girls^8^. While genetic etiology of the disorder is established and behavioral phenotype is well described, neurophysiological level of dysfunction is not well understood, as no crucial abnormalities that can explain such drastic behavioral problems is still identified. At the same time, objective neurophysiological biomarkers of RTT severity are of high importance e.g. to serve as an output measure for clinical trials that run for this disorder.

Previous studies showed several abnormalities in spontaneous EEG in RTT (for review see^5^). Here we plan to verify them in an independent sample of girls with RTT as well as to examine the long-range temporal correlation in this disorder that was never done before.

While Rett Syndrome incidence is 1:10,000–20,000, understanding its pathophysiology sheds light into the fundamental question of the mechanistic link between genetic mutations, brain processes and behavior. Moreover, MECP2 path is disturbed in a sufficient number of patients with autism spectrum disorders^9^ and, thus, establishing the neurophysiological marker of RTT severity will help to better understand and treat these disorders as well.

## Method

### Participants

Children with Rett syndrome (RTT, n = 23, 3.7–17.1 years, M = 9.1, SD = 4.1 years, all females) and typically developed children (TD, n = 38, 3.04–16.9 years, M = 9.8, SD = 3.3 years, 27 females) participated in the experiment. RTT patients were recruited during clinical visits to the Research Clinical Institute of Pediatrics in Moscow, Russian Federation. The diagnosis was based on current diagnostic criteria^7^ and was confirmed clinically by a medical doctor specializing in this population (V.V.) as well as via genetic testing (all with *MECP2* abnormalities). Severity of RTT was measured using the Rett Syndrome Severity Scale (RSSS)^10^. History of seizures were reported for 9 RTT patients from our group.

Parents or legal guardians have given written informed consent to their children's participation in the study, after the procedure was explained to them. Children have given verbal consent to participate and where possible, assent from the patient was also ascertained. The research procedure was approved by the ethical committee of Institute of Higher Nervous Activity and Neurophysiology, Russian Academy of Science, IHNA and Nph RAS (protocol №2 on April 30th, 2020). All aspects of the research conformed to the tenets of the Declaration of Helsinki.

### EEG recording

The EEG recording from 23 channels (10–20 scheme) was organized in blocks of 30 s recordings with alternating eyes open, passive hand movement and no visual input condition. As RTT patients are hard in following instructions, for all children we used an airplane mask for no visual input condition to simulate fixation-off condition. Here we focus on EEG from the block with no visual input (epoched of 30 s, n = 3–9). Raw EEG signals were filtered in the range of 1–40 Hz using FIR filter, and independent component analysis (ICA) was used to remove artifacts (Fieldtrip toolbox^11^). Additionally, ICA decompositions were examined for artifacts using the open-source Crowdsourcing Platform for Automatic Labeling of Independent Components in Electroencephalography (ALICE^12^, http://alice.adase.org/). Finally, all signals were re-referenced to the common reference.

### Spectral analysis

We calculated wavelet power (WP)

$$WP\left( t \right) \, = \left| {\mathop \smallint \limits_{F}^{{}} \left| {W\left( {f,t} \right)} \right|^{2} df,\,\,\,W\left( {f,t} \right) = \sqrt f \mathop \smallint \limits_{ - \infty }^{\infty } x\left( t \right)\psi^{*} \left( {f\left( {t^{\prime } - t} \right)} \right)dt^{\prime } } \right.,$$

where $x\left( t \right)$ is the raw EEG signal, *F* = 4–40 Hz is the frequency band of interest, $\psi \left( \eta \right)$ is the Morlet wavelet

$$\psi \left( \eta \right) = \frac{1}{{\sqrt[4]{\pi }}}e^{{ - \eta^{2} /2\sigma^{2} + j2\pi f\eta }} ,$$

$j = \sqrt { - 1} ,$ an asterisk indicates a complex conjugation, and $\sigma = n/2\pi f$. The number of cycles, $n$, depended on the signal frequency, $f$, as $n = f$. For each subject we averaged WP over time and over the epochs. To minimize between-subject variability, we considered normalized wavelet power (NWP) by contrasting WP at each sensor/frequency to the WP averaged over all sensors and all frequencies. All calculations were performed using the Fieldtrip toolbox in MATLAB.

### Long-range temporal correlations

Long-range temporal correlations (LRTC) were assessed by detrended fluctuation analysis (DFA). This parameter estimates the statistical self-affinity of the signal. After the preprocessing procedure, all epochs were loaded to the Neurophysiological Biomarker Toolbox (NBT, http://www.nbtwiki.net/) and we strictly followed the pipeline, described by Hardstone and colleagues for DFA calculation^13^. First, we filtered the signal in the range of 6–13 Hz using a FIR filter and got the amplitude envelope using the Hilbert transform. Following recommendation, the filter order was automatically set by the NBT toolbox ensuring that at least two 6 Hz oscillations cycles were covered by the filter window. The fluctuations were calculated in the frequency band of 6–13 Hz, using 50% overlapping windows from 0.8 to 30 s, and the DFA exponent was found by fitting from 2 to 15 s. For each subject, we averaged the DFA exponent over all epochs and days.

### Statistical testing

To contrast NWP and DFA between RTT and TD groups, we used an unpaired t-test in conjunction with the nonparametric cluster-based correction for the multiple comparisons and the Monte-Carlo randomization. Elements that passed a threshold value corresponding to a p-value of 0.001 were marked together with their neighboring elements and collected into separate negative and positive clusters. The minimal number of required neighbors was set to 0 (a single channel could be considered as a cluster). A cluster was significant when the p-value was below 0.025, corresponding to a false alarm rate of 0.05 in a two-tailed test, as we separately examined the hypothesis on the RTT < TD (negative cluster) and RTT > TD (positive cluster). The number of permutations was 5000. Analysis was performed in the Fieldtrip toolbox for MATLAB.

As EEG pattern changes with age we examine the relation of the defined EEG-parameters with age by means of two-tailed Pearson correlation. Taking into account the direction of alterations revealed by group comparison, we examined the link between EEG measures and clinical manifestation of the disorder by one-tailed partial Pearson correlation, controlling for age. A Mann–Whitney test was performed to examine the difference between RTT patients with and without history of seizures.

A logistic regression was performed to ascertain the effects of significant clusters formed at the previous stage of analysis on the likelihood that the child has Rett syndrome (RTT). We used ROC-AUC and fivefold cross-validation to evaluate the model’s ability to distinguish between RTT (label: 1) and TD (label: 0) groups.

## Results

### Spectral analysis

**Figure 1:**
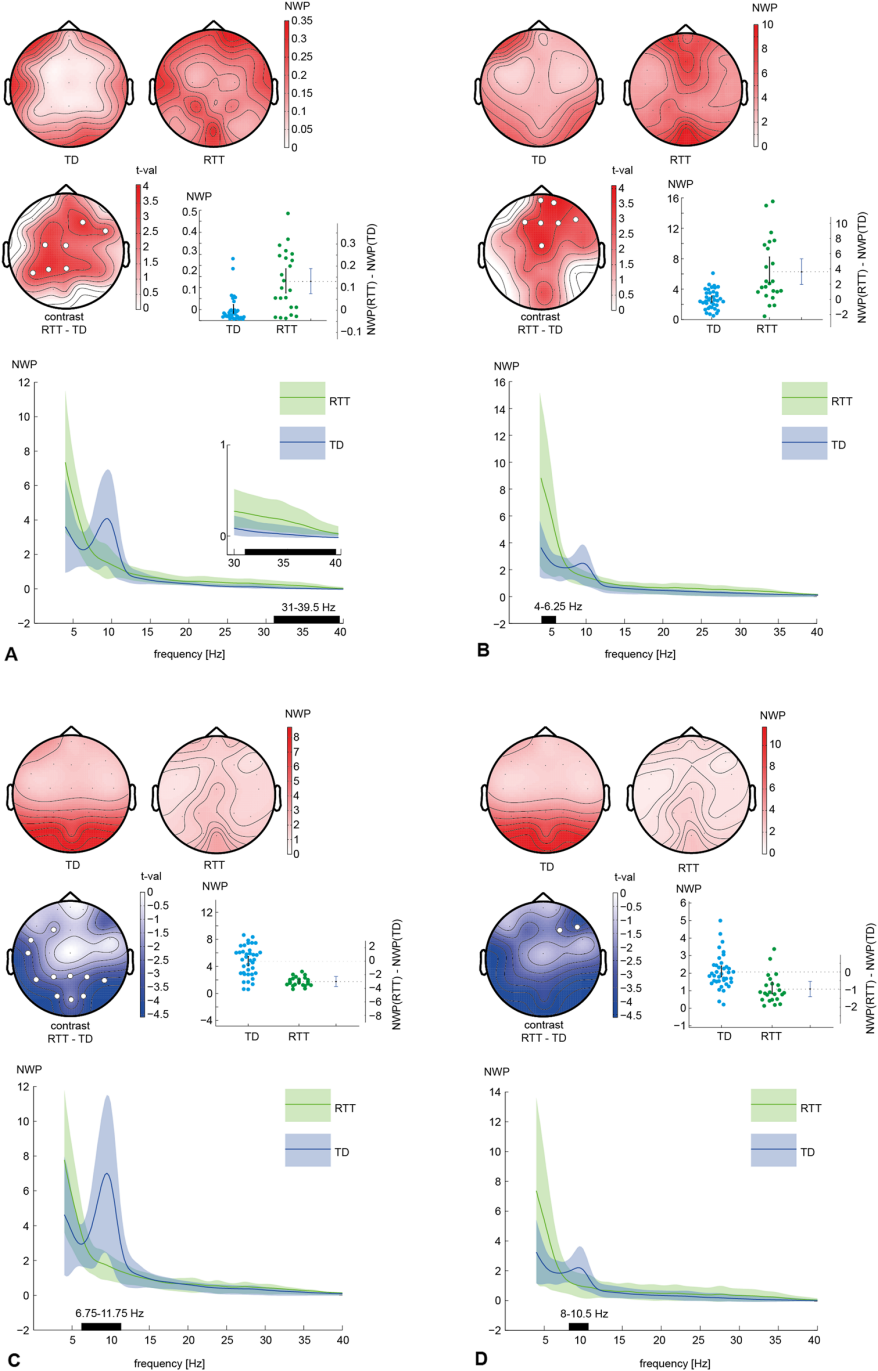
Difference in EEG spectra between RTT and TD. For each significant cluster you can see: at the first row—topographic maps, that show the normalized wavelet power (NWP) in the RTT and TD groups (group mean), at the second row the t-values as the result of statistical testing the NWP difference between groups with electrodes included in the cluster marked by white circles. Darkness of colors in topographic maps represents the increase in absolute values (blue—towards negative values, when RTT’s values are smaller than TD's, and red—towards the positive ones, when RTT’s values are smaller than TD's). Also second row contains the estimation plots that illustrate NWP for the RTT and TD groups (mean, standard errors are represented by black vertical line and each dot corresponds with individual values: green for RTT, blue for TD) as well as the mean NWP difference between them with the 95% CIs (third vertical line). The last third row contains the NWP over the whole spectrum average over the electrodes from the cluster (green line for RTT and blue for TD, shading for 95% CIs) with significant between group differences highlighted by the horizontal black line at x-axis. Here we revealed four clusters of significant differences: The NWP in the RTT was larger than TD groups in gamma, 31–39.5 Hz (**A**) and theta, 4–6.25 Hz (**B**) bands, and smaller in alpha band, 6.75–11.75 Hz (**C**) and 8–10.5 Hz (**D**).

Contrasting normalized wavelet power (NWP) between RTT and TD groups with *p* = 0.001, we found two positive and two negative clusters, where the NWP was significantly increased or decreased for patients (Fig. 1). The first positive cluster (further called gamma-cluster, Fig. 1A) with *p* = 0.0008 appeared in the frequency band of 31–39.75 Hz and included EEG sensors F4, C3, Cz, P7, P3, Pz, FT8. In this cluster, NWP for the TD group (0.047 [95% CIs 0.033 0.072]) was lower than in the RTT group (0.178 [95% CIs 0.125 0.235]): RTT—TD = 0.131 [95% CIs 0.075 0.188]. The second positive cluster (Fig. 1B) with *p* = 0.002 appeared in the frequency band of 4–6.25 Hz and included EEG sensors Fp2, F3, Fz, F4, F8, Cz, Fpz. In this theta cluster, NWP for the TD group (2.67 [95% CIs 2.293 3.103]) was lower than for the RTT group (6.276 [95% CIs 4.817 8.227]): RTT—TD = 3.606 [95% CIs 1.95 5.325]. The first negative cluster (Fig. 1C) with *p* = 0.0002 appeared in the frequency band of 6.75–11.75 Hz and included EEG sensors F3, T7, P7, P3, Pz, P4, P8, O1, O2, Oz, FT7. In this further called alpha1 cluster NWP in the TD group (4.765 [95% CIs 4.082 5.467]) exceed NWP in the RTT group (1.756 [95% CIs 1.502 2.039]): RTT—TD = − 3.009 [95% CIs − 3.736 − 2.262]. The second negative cluster (Fig. 1D) with *p* = 0.0128 appeared in the frequency band of 8–10.5 Hz and included EEG sensors F4, F8. In this alpha2 cluster NWP in the TD group (2.07 [95% CIs 1.798 2.412]) exceed NWP in the RTT group (1.094 [95% CIs 0.825 1.495]): RTT—TD = − 0.975 [95% CIs − 1.408 − 0.537].

### Long-range temporal correlations

**Figure 2:**
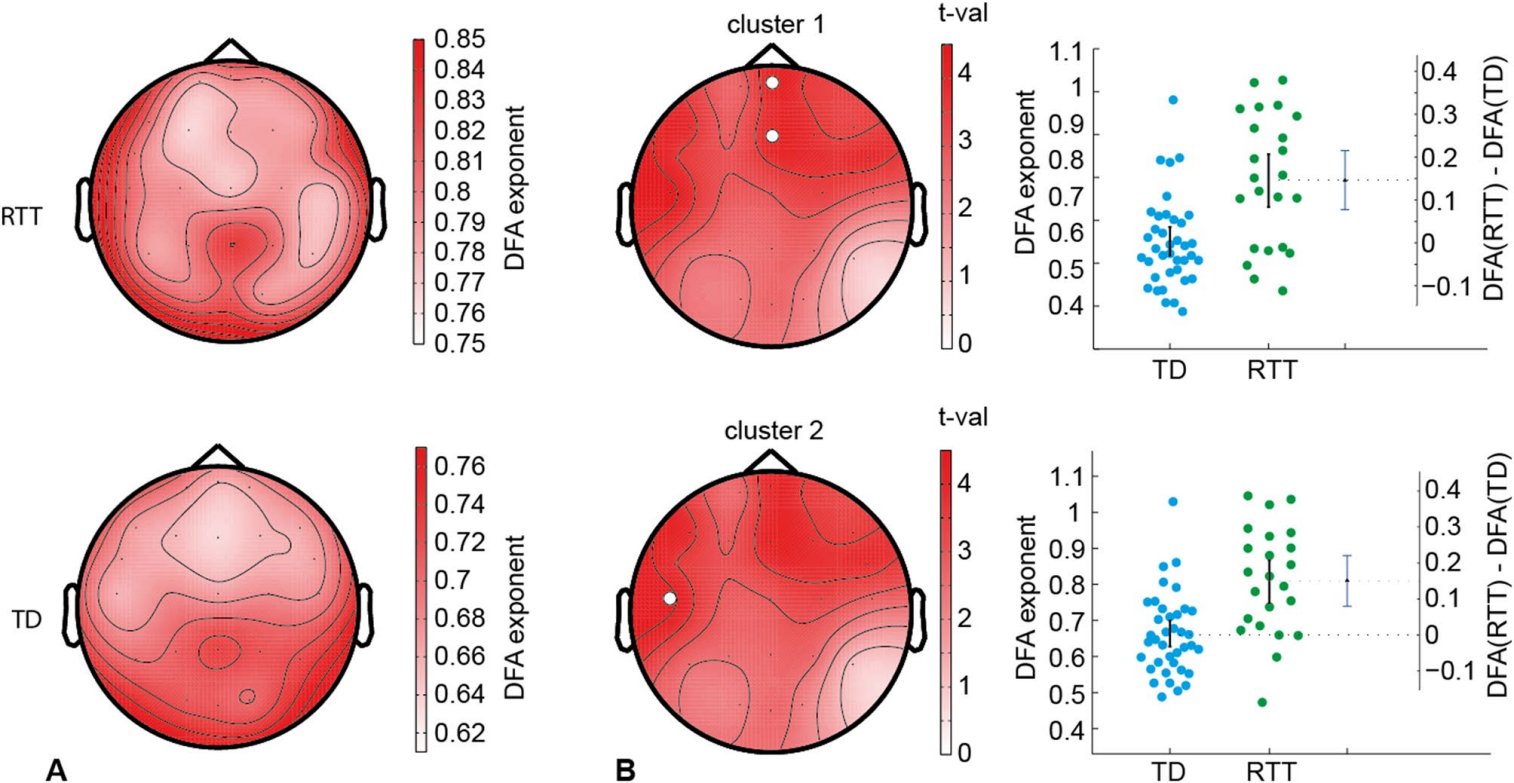
Difference in the DFA exponent between RTT and TD. (**A**) Topographic maps show DFA exponent in the RTT and TD groups (group mean). (**B**) Topographic maps reflecting t-values as the result of statistical testing the DFA difference between RTT and TD groups. Two significant clusters were revealed with electrodes included in the cluster marked by white circles. Darkness of colors in topographic maps represents the increase in absolute values. Corresponding estimation plots are shown at the right. mean DFA for the RTT and TD groups (mean, standard errors are represented by black vertical line and each dot corresponds with individual values: green for RTT, blue for TD) as well as the mean DFA difference between them with the 95% CIs (third vertical line).

DFA exponent showed different topographic profiles in the RTT and TD groups (Fig. 2A). In the RTT group, there was a local increase in DFA in the frontal and temporal EEG sensors bilaterally. The TD group demonstrated high DFA in the frontal midline electrodes. Two positive clusters were observed (Fig. 2B). The first cluster with *p* = 0.0007 includes EEG sensors Fz and Fpz. The mean DFA in the RTT group (0.793 [95% CIs 0.732, 0.853]) was higher than in the TD group (0.647 [95% CI 0.618, 0.687]): DFA(RTT) – DFA(TD) = 0.145 [95% CI 0.076, 0.214]. The second cluster with p = 0.001 included EEG sensor T7. The mean DFA in the RTT group (0.810 [95% CI 0.749, 0.869]) was higher than in the TD group (0.659 [95% CI 0.627, 0.699]): DFA(RTT) − DFA(TD) = 0.151 [95% CI 0.08, 0.217].

### EEG characteristics for group classification

**Figure 3:**
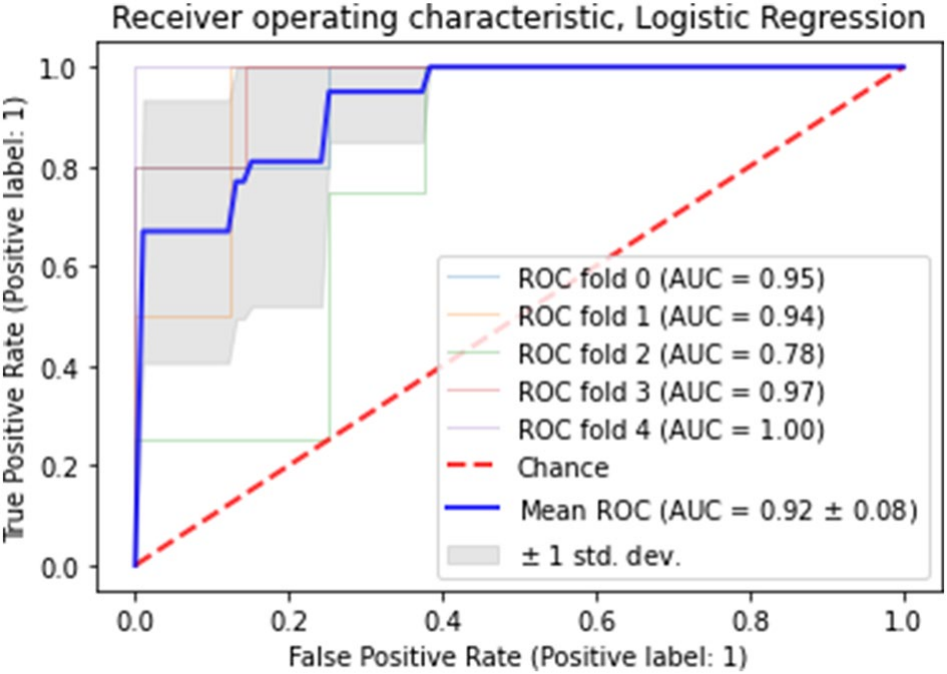
Results of the model’s evaluation using ROC-AUC and a fivefold cross-validation.

The logistic regression model was statistically significant, chi2(5) = 62.535, *p* < 0.001. The model explained 85.5% (Nagelkerke R2) of the variance in the Rett syndrome and correctly classified 91.8% of cases. Figure 3 shows ROC curves for each fold with the mean ROC (solid blue line) and standard deviation (a gray area). The dashed line corresponds to the chance level. AUC varied from 0.78 to 1.00 between the folds achieving a mean value of 0.92 ± 0.08.

### Relationship between neurophysiological and clinical measures

**Figure 4:**
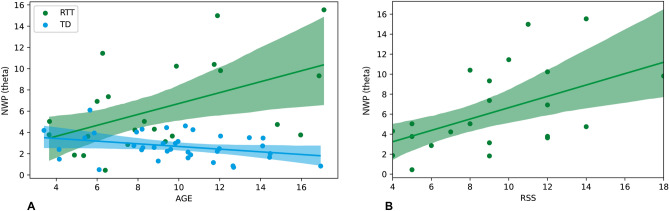
Correlation of theta NWP with age (**A**) and RTT severity (**B**). Dots represent individual values (blue for TD and green for RTT), shaded color corresponds with 95% CIs.

Among our EEG measures only theta cluster correlated with age in RTT (r(23) = 0.51, *p* = 0.014), pointing to the worsening of the condition with time. As can be seen in Fig. 4A, theta cluster activity did not change with age in typically developing children, pointing to the increasing difference between RTT and TD with age. However, even when the effect of age was partialed out, the increase in theta power over fronto-central regions positively correlated with severity of RTT clinical manifestation (r(20) = 0.42, *p* = 0.026, Fig. 4B). No other EEG parameters showed significant relation with RSS, when taking into account the direction of changes. No significant differences were found between RTT patients with and without history of seizures for any significant clusters.

## Discussion

This paper examined EEG properties of spontaneous EEG recorded in no visual input condition in search for objective neurophysiological markers of RTT as well as its severity. We confirmed some previous findings of a few previous quantitative EEG studies such as increased theta and decreased alpha power in RTT^14–16^, supporting general slowing of background activity in RTT also frequently reported in clinical assessment of these patients (review^5^). Moreover, our data support the relationship of the increased theta power with patients’ age and RTT symptoms^17,18^. While relation of the theta increase with the progression of the RTT symptoms is important finding, it also points to its potential low power to detect RTT before the main symptoms are clearly manifested, e.g. in the pre-regression stage. Indeed Roche and colleagues found that theta power is even decreased in very young RTT patients in the active regression stage^16^. Taking into account that predominance of low frequency activity in EEG is not specific to RTT and characterizes a number of other neurological conditions and neurodevelopmental disorders^19–21^, this potential biomarker of RTT severity might be related to general deterioration of brain function.

Gamma activity was examined only in two previous studies of patients with RTT, however, the experimental conditions did not match. One study^22^ looked at gamma activity during slow wave sleep and suggested increased cortical excitability as no typical decrease in gamma power was reported from 2–5 to 6–9 years of age. Another study^16^ examined gamma band response during video watching and did not find any significant results in spite of one of the largest sample of RTT girls examined (n = 57). Our study showed increased gamma activity during no visual input condition, suggesting increased cortical excitability. Specificity of this result to no visual input condition needs to be additionally examined in the future studies. Among other disorders, gamma band activity was shown to be increased in ASD, epilepsy, being indicative of higher excitation/inhibition ratio in neuronal activity. At the same time, interpreting the gamma band response in EEG should be made with caution as it might reflect muscle activity, which overlaps in frequencies with cortical gamma oscillations^23^. However, as the gamma cluster in our study extends also into the central region this interpretation seems to be inapplicable to our results.

Long-range temporal connectivity provides the measure of scale-free properties of EEG signal, also related to the excitation/inhibition balance. Increased DFA coefficient in RTT suggests non-optimal state of neuronal networks that have more predictable activity. This novel result for RTT fits well with previous reports in neurodevelopmental disorders, such as idiopathic ASD^24^ as well as in patients with STXBP1 syndrome^25^ that have increased DFA as well. Among common features of these disorders is comorbidity with epilepsy, which is also characterized by increased DFA measure with intracranial recordings near the epileptogenic region^26,27^. However, ASD with visually detected epileptiform activity showed smaller DFA than ASD without evident EEG abnormalities, suggesting some compensatory mechanism^24^. Noteworthy, that LRTC of hemoglobin measured using fNIRS were smaller in idiopathic ASD^28^, pointing to heterogeneity of ASD.

Overall, parameters of EEG recorded during several blocks of 30-s no visual input condition allowed segregating RTT from TD with high accuracy (ROC AUC = 0.92 ± 0.08), pointing to their clinical relevance.

As Rett syndrome affects mostly girls and our control group was more representative of the general population and also included males (about 30%), it is important to discuss the potential effect of biological sex on our findings. Surprisingly little is known about resting EEG differences in males and females. Maturational lag was reported in girls compared to boys with their theta power being higher and alpha power being lower than in boys^29^. However, the sex-related differences decreased with age in this study. Recent study from the same research group^30^ confirmed increased theta, but reported also increased alpha power in females compared to males in young adulthood. In our study theta power was not only increased in girls with RTT, but was also related to Rett Syndrome severity in patients, thus supporting its clinical relevance. Higher gamma-band activity was also observed in adult females as compared to males^31^. While this sex-effect might increase the RTT vs. control difference, it is clearly not a primary factor as seen from the data distribution with TD values largely grouped together and separated from most RTT’s measures. Previous study^32^ of long-range temporal correlations reported the larger DFA in males than in females. Thus, sex-effect here might partially counteract our effect of atypically high DFA in girls with RTT decreasing the between group difference that might in fact be even larger.

Among the limitations of our study is the absence of clinical control groups with other neurodevelopmental disorders to examine specificity of the obtained pattern of differences to girls with Rett syndrome. It would be also preferred to compare EEG parameters with and without visual input, although for this particular recording block there were no so-called “resting state” eyes open conditions. Important direction for the future work would be longitudinal follow-up of the EEG and behavioral changes in girls with RTT for more direct assessment of the link between neurophysiological and clinical symptoms.

## Data Availability

Anonymized data are available upon request from the corresponding author.
